# The multi-sectorial emergency response to a cholera outbreak in Internally Displaced Persons camps in Borno State, Nigeria, 2017

**DOI:** 10.1136/bmjgh-2019-002000

**Published:** 2020-01-28

**Authors:** Moise Chi Ngwa, Alemu Wondimagegnehu, Ifeanyi Okudo, Collins Owili, Uzoma Ugochukwu, Peter Clement, Isabelle Devaux, Lorenzo Pezzoli, Chikwe Ihekweazu, Mohammed Abba Jimme, Peter Winch, David A Sack

**Affiliations:** 1 International Health, Johns Hopkins University Bloomberg School of Public Health, Baltimore, Maryland, USA; 2 World Health Organisation, Nigeria Country Office, Abuja, Nigeria; 3 World Health Organization, Maiduguri, Nigeria; 4 World Health Organization, Geneve, Switzerland; 5 Nigeria Centre for Disease Control, Abuja, Nigeria; 6 Geography, University of Maiduguri Faculty of Social Science, Maiduguri, Nigeria

**Keywords:** qualitative research, cholera, emergency response, Borno State, Nigeria, reactive oral cholera vaccine, internally displaced persons camp, monitoring and evaluation

## Abstract

**Introduction:**

In August 2017, a cholera outbreak started in Muna Garage Internally Displaced Persons camp, Borno state, Nigeria and >5000 cases occurred in six local government areas. This qualitative study evaluated perspectives about the emergency response to this outbreak.

**Methods:**

We conducted 39 key informant interviews and focus group discussions, and reviewed 21 documents with participants involved with surveillance, water, sanitation, hygiene, case management, oral cholera vaccine (OCV), communications, logistics and coordination. Qualitative data analysis used thematic techniques comprising key words in context, word repetition and key sector terms.

**Results:**

Authorities were alerted quickly, but outbreak declaration took 12 days due to a 10-day delay waiting for culture confirmation. Outbreak investigation revealed several potential transmission channels, but a leaking latrine around the index cases’ house was not repaired for more than 7 days. Chlorine was initially not accepted by the community due to rumours that it would sterilise women. Key messages were in Hausa, although Kanuri was the primary local language; later this was corrected. Planning would have benefited using exercise drills to identify weaknesses, and inventory sharing to avoid stock outs. The response by the Rural Water Supply and Sanitation Agency was perceived to be slow and an increased risk from a religious festival was not recognised. Case management was provided at treatment centres, but some partners were concerned that their work was not recognised asking, ‘Who gets the glory and the data?’ Nearly one million people received OCV and its distribution benefited from a robust infrastructure for polio vaccination. There was initial anxiety, rumour and reluctance about OCV, attributed by many to lack of formative research prior to vaccine implementation. Coordination was slow initially, but improved with activation of an emergency operations centre (EOC) that enabled implementation of incident management system to coordinate multisectoral activities and meetings held at 16:00 hours daily. The synergy between partners and government improved when each recognised the government’s leadership role.

**Conclusion:**

Despite a timely alert of the outbreak, delayed laboratory confirmation slowed initial response. Initial responses to the outbreak were not well coordinated but improved with the EOC. Understanding behaviours and community norms through rapid formative research should improve the effectiveness of the emergency response to a cholera outbreak. OCV distribution was efficient and benefited from the polio vaccine infrastructure.

**Key questions**

**What is already known?**

- Boko Haram conflict displaced thousands into unsanitary internally displeased persons (IDPs) camps in Borno, and cholera outbreak in Muna Garage IDPs camp affected 5340 cases (61 deaths).

**What are the new findings?**

- Authorities were alerted quickly, but outbreak declaration took 12 days due to a 10-day delay waiting for culture confirmation.
- Outbreak investigation revealed several potential transmission channels in the camp, but a leaking latrine around the index cases’ house was not repaired for more than 7 days.
- Language, coordination and vaccine hesitancy all changed with IDP camp stratification, activation of emergency operations centre and occurrence of cholera death in community, respectively.

**What do the new findings imply?**

- There is need to strengthen laboratory surveillance in Borno, improve water, sanitation and hygiene conditions in IDPs camps, and conduct formative research as part of risk assessment to inform interventions including use of oral cholera vaccine.
- Community engagement should precede community entry to facilitate buy-in to technical and behavioural innervations.

## Introduction

Cholera claims about 1.3–4.0 million cases (21 000–143 000 deaths) annually worldwide,^1^ in settings with poor access to water, sanitation and hygiene (WASH), including Nigeria. The risk of cholera is considered high in humanitarian crises, be it man made (conflict/war) or natural (droughts/floods), with massive population movements into internally displaced persons (IDPs) camps often without adequate WASH and health facilities.^2^ Recently, humanitarian crises linked with conflict caused explosive cholera outbreaks in Yemen, South Sudan, Iraq and Somalia^3^ while between 1990 and 2010, 1 in every 3 droughts and 15 floods caused outbreak in sub-Saharan Africa.^4^ Given the challenges associated with restoring disrupted WASH systems and health facilities to vulnerable populations in IDPs camps and other conditions, the oral cholera vaccine (OCV) has been implemented as part of comprehensive measures to cholera control in endemic,^2^ outbreak and humanitarian crises settings.^5^ Quantitative studies have looked into OCV interventions in IDPs camps.^6–10^ However, excepting Spiegel *et al,* 11 there is scarcity in qualitative comprehensive analysis of cholera emergency response measures in humanitarian crises setting with focus on IDPs camps.

**Figure 1:**
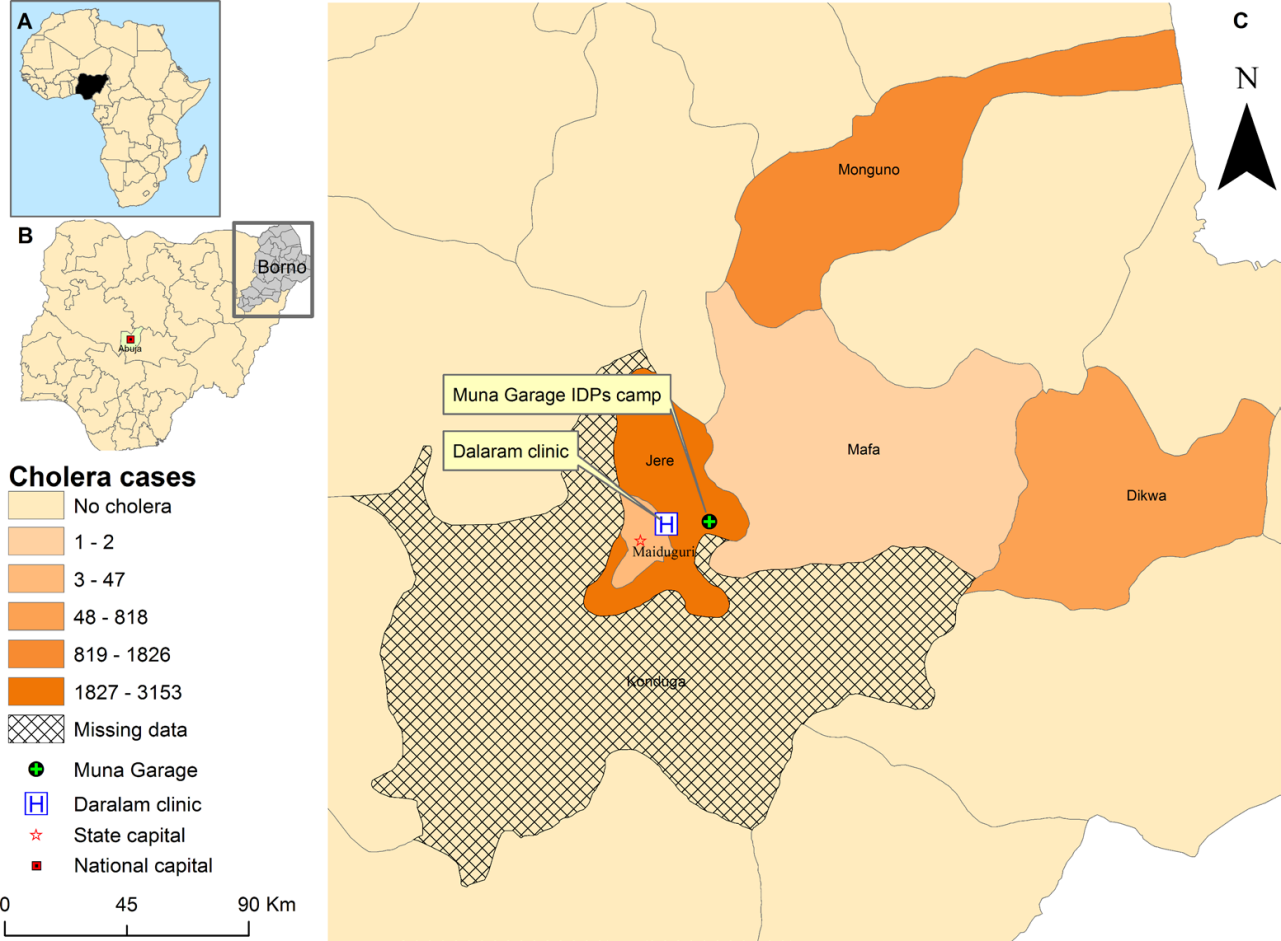
Spatial occurrence of cholera in Borno State, 2017. (A) shows Nigeria within Africa while (B) portrays national capital of Nigeria (Abuja) and Borno State, and (C) depicts the six LGAs affected by the 2017 cholera outbreak (LGA is equivalent to a health district). The index case was detected in Muna Garage Internally Displaced Persons (IDPs) camp and the first cases where transported by ambulance to Dalaram clinic for treatment (insert C). The shapefile was obtained from WHO Nigeria Country Office as part of document review. LGA, local government area.

**Figure 2:**
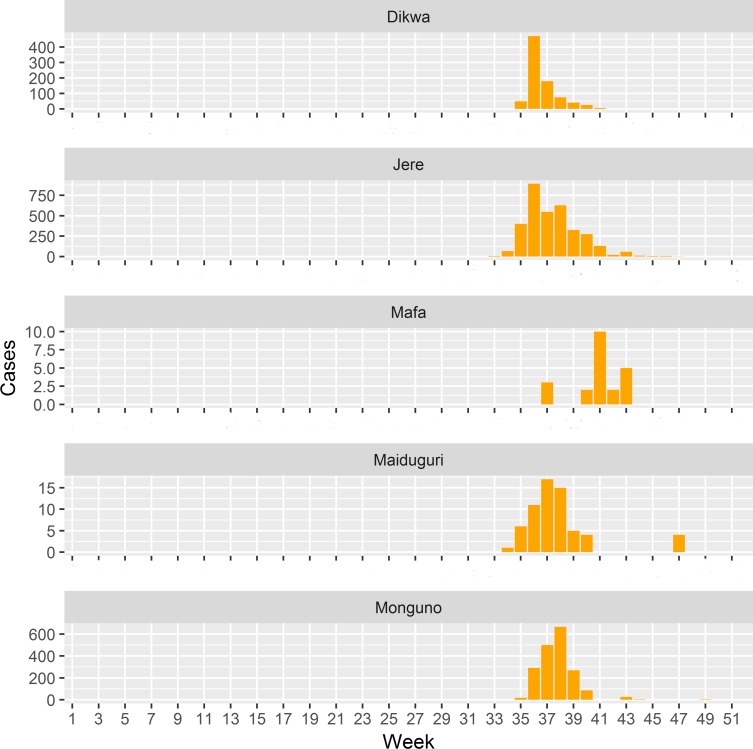
Weekly occurrence of cholera in Borno State, 2017. Konduga data were missing. The outbreak started in Jere (week 33) then spread to Maiduguri (week 34) and to Monguno and Dikwa (week 35), and finally to Mafa (week 37). The data were obtain from surveillance line list as part of document review.

Cholera was first reported in Nigeria (figure 1A,B) in 1970.^2^ Between 1991 and 2018, a total of 321 148 cases (18 644 deaths) were reported across Nigeria.^12 13^ In 10 years of Boko Haram attacks,^14^ which has left an estimated 20 000 people dead and displaced 2.6 million in the northeast with insufficient food and clean water,^15^ Borno state has seen an upsurge of people being displaced into 1 of 164 IDPs camps including 59 at high risk of floods.^16^ In 2010, flooding preceded peaks of outbreak (21 111 cases, case fatality ratio (CFR) 5.1%) that started in local government areas (LGAs) in Borno.^17^ In August 2017, another outbreak started in Borno, in the Muna Garage IDP camp (figure 1C). By October 2017 (figure 2), it affected five other LGAs (figure 1C) with circa 5340 cases (CFR 1.14%). In response, the Nigeria government through the Borno Ministry of Health (BMOH) and international partners implemented comprehensive cholera control measures including OCV to stop the outbreak. As part of monitoring and evaluation to inform future emergency response effort, we investigated perspectives of government and partners on the outbreak emergency response.

## Methods

### Study setting

In Abuja, Nigeria capital, interviews and discussions were held in WHO Nigeria, Nigeria Center for Disease Control, Federal Ministry of Water Resources (FMWR) and Nigeria Primary Health Care Development Agency field offices. In Borno, they were conducted in WHO and United Nations Children’s Fund (UNICEF) offices in Maiduguri and emergency operations centre (EOC) in Jere (figure 1B,C).

Participants were recruited using purposive and snowball sampling strategies.^18^ Key informants (KIs) and group discussion participants were required to be representatives of organisations who took active part in the emergency response. KIs were participants without location and time flexibility such as top-ranking officials at BMOH and WHO while group discussion participants ranged from 3 to 7 members who had location and time flexibility. In Abuja, Maiduguri and Jere a purposeful sample of participants were initially selected from WHO offices and then extended outward to include respondents from other organisations. In each location, we obtained a list of organisations and recruited their representatives in person or email or phone. Next, the recruited participants referred us to other partner organisations, who, in turn, recommended further contacts. The recruitment ended when participants could no longer recommend any new organisations.

### Patient and public involvement

Patients or the public were not involved in the development or implementation of this study.

### Data collection

Qualitative data were collected from February 19 to 28 2018 incorporating document review, key informant interview (KII) and focus group discussion (FGD) to understand the perspectives. Two members, one interviewer (PhD researcher with training and experience in qualitative methods) and an assistant (note taker) with training in field note taking, but without formal training in qualitative methods collected data. Documents reviewed included geographic information system shapefile and maps, OCV microplanning guides, cholera line list and weekly situation report, draft schedule of response activities, team formation documents and reports of meeting minutes. During KII and FGDs, the interviewer led the interviews, moderated the group discussions and audio typed the conversation(s) using a SONY audio (voice) recorder while the assistant took written notes; where participants elected to be anonymous we took only written notes. We developed interview guides by thematic pillar, namely: (1) Health (surveillance: epidemiology/laboratory, case management and vaccination); (2) WASH and (3) Crosscutting (risk communication, coordination and logistics) that contained open-ended questions (see online supplementary S1) to get the perspectives. The guide was not pretested. During FGDs, participants were encouraged to expresses their views in their own vocabulary and explore further themes of interest to them. The duration of KIIs ranged from 30 to 45 min and from 1 to 2 hours for FGDs. The data were encrypted and stored at Johns Hopkins University in a secure database for analysis.

10.1136/bmjgh-2019-002000.supp1 Supplementary data

### Data analyses

Tape-recorded interviews, discussions and field notes were transcribed into English. In the transcriptions, important aspects of data interpretation such as voice speed, tone and points of stress were captured. The transcripts were arranged according to government and partner data. Focus groups and individual interviews were sorted and coded hierarchically within the thematic pillars and then triangulated with the document review data. Data coding and analysis were performed using pen and paper.

Further, verbal informed consent was obtained prior to conducting any interviews/discussions and after explaining the purposes of the study.

## Results

Evaluation of the emergency response included perspectives from 17 government and 22 partner representatives as well as document review. Analytical perspectives emerging were grouped along three thematic pillars.

### Health

#### Outbreak detection, notification/reporting

Three surveillance systems were used in communicating the outbreak at various levels. These were the Early Warning Alert and Response System (EWARS),^19 20^ Integrated Disease Surveillance and Response (IDSR)^21 22^ and phone platform (alerts by mobile phones). At state level, phone platform reported the index case, but disagreements arose as to who reported the case:

On August 16th, 2017, we got a call from Médecins Sans Frontières (MSF) telling us about a case of acute watery diarrhea, which prompted an immediate visit to their facility in Gwange (WHO official).

BMOH agreed to have received an alert from MSF about the index case:

The outbreak started on a site supported by MSF and they ‘alerted’ my office through the health sector coordination structure… (Government official).

From the two excerpts above, the index was reported by MSF. Yet, not all agreed:

A case was admitted in a supportive health care facility managed by UNICEF in Muna Garage and we got the alert notification by phone on Friday August 18, 2017 (Nongovernmental Organization (NGO) official).

The phrase ‘health sector coordination structure’ shows the utility of the phone platform in the disease notification. Therefore, the phone platform was very critical at the early stages of the outbreak.

At the national level, the outbreak was reported through routine surveillance and EWARS systems:

We were notified through the routine surveillance system as we saw reports stating cases were coming out from IDP camps (Government official).

What is mentioned above as routine surveillance refers to IDSR strategy.^23^ Meanwhile the WHO Country Office was notified through EWARS. In surveillance, the word ‘alert’ portrays emergency and urgency to respond, but the word ‘notify’ was used more frequently, which does not signal response urgency.

#### Outbreak confirmation and declaration

Very critical to the emergency response was an outbreak declaration, which depended on rapid diagnostic tests (RDTs) and confirmation by laboratory culture. In this case, a positive RDT from a stool sample from an index case was reported as negative by culture.

…the MSF call on August 16, 2017 prompted an immediate visit to their facility. We conducted RDTs in the facility, which turned out positive. Then, we sent part of the sample to the University of Maiduguri Teaching Hospital (UMTH) lab for confirmation, which turned out negative to culture (WHO official).

Lack of laboratory capacity at the University of Maiduguri Teaching Hospital (UMTH) laboratory such as reagents and trained lab technicians contributed to the negative culture results as the same samples were found to be culture positive for *Vibrio cholerae* in the reference labratory in Lagos.

…But because we saw the stool that was classical of cholera, we sent part of the sample to the reference laboratory in Lagos, which turned out positive to culture (WHO official).

A positive culture from Lagos meant delays in laboratory confirmation of the clinical diagnosis and RDT result, which, in turn, delayed outbreak declaration. Nonetheless, BMOH disagrees there was delay in outbreak declaration, stating:

Yes, the outbreak was declared timely because the issue of diarrhea and vomiting are all year round in this part of the country… So when we had these cases…we thought it was just normal treatment of diarrhea and vomiting and so on. But when the cases were becoming alarming, and the first confirmation on 26th August, and by 28 of August, we alerted the highest authority, the Honorable Commissioner of Health, to declare the outbreak so that we can have synergy among all partners in the State. So he declared the State as having outbreak of cholera (Government official).

Declaring the outbreak was critical to have synergy in response among partners. Still, declaring the outbreak was not a health issue only; it was linked with political considerations:

We also had to take into cognizance the political situation and repercussions of declaring an outbreak. We are in democracy. Somebody will just think that you are trying to score political points by declaring cholera outbreak. We had to be convinced and… see reason to declare an outbreak (Government official).

However, as the outbreak started on 16 August, was confirmed on the 26 (10 days later), and officially declared on 28 August (12 days after), several partners stated that outbreak declaration was delayed. Further, they felt, given there was positive RDT result and clinical evidence of transmission, reluctance to declare outbreak was attributed to vacation and timing of the annual Muslim pilgrimage to Mecca (declaring outbreak would have prevented local Muslim officials from travel to Mecca). Others felt that the WHO official policy of declaring cholera outbreak only after culture confirmation is ill advised in settings where laboratory capacity is rare. They suggested that positive RDTs and clinical evidence of transmission should suffice to declare cholera outbreak. The declaration of the outbreak 12 days after onset likely slowed response and contributed to its rapid spread from Muna Garage IDP camp to six other LGA (figure 1C).

#### Outbreak investigation and spread

WHO Surveillance Team traced the index case back to his household and found sewage leakage from one of the latrines flowing into his household.

When we went to the index case’s house, we saw sewage that was leaking into his house and we notified the State-WASH sector namely Rural Water Safety and Sanitation Agency (RUWASSA) and Borno State Environmental Protection Agency for repairs. But when we returned one week later to decontaminate the leaking facility, it was still unrepaired (WHO official).

Government agencies may have underestimated the magnitude of the situation. This excerpt illustrates that the 2017 Muna Garage cholera outbreak may have originated from a leaking latrine. Within a week cholera spread to the whole camp and host communities probably amplified by a festival.

The outbreak spread rapidly, because there was this festival with lots of eating and celebration and after which there was a jump in the number of cases. So we knew we had a full-blown outbreak (WHO official).

The festival above refers to ‘Eid El Kabir’, a Muslim religious festival that entails lots of movement and food sharing, which coincided with the outbreak onset and likely exacerbated its transmission and spread. Though the outbreak started in Muna Garage camp, no cholera treatment centres (CTCs) were immediately constructed in the camp. Rather patients were sent by ambulance to Dalaram Clinic (figure 1C) in Old Maiduguri for treatment.

Basically, this disease spread out of Muna Garage camp as a result of lack of CTCs there at the early phase of the outbreak. The thinking was to manage the outbreak with a Primary Health Care facility, Dalaram Clinic, some few Km away from Muna Garage (NGO official).

A WHO official echoed this account:

The issue was disagreement between BMOH and partners in selecting the site for the second CTC. BMOH wanted it within the camp while the other partners wanted a little bit further. This definitely delayed the CTC construction (WHO official).

Transporting cholera patients from Muna to Dalaram Clinic was risky and made case management difficult.

### Case management

Case management achieved a CFR of 1.14%, the lowest ever in Borno. After outbreak declaration, patients were treated in one or all of three facilities including oral rehydration point (ORP) or cholera treatment unit (CTU) or CTC. However, these were confusing to patients.

… MSF set up an oral rehydration point close to the UNICEF cholera treatment center, which became conflicting as the locals didn’t know the difference between acute watery diarrhea and cholera (NGO official).

The setting up of the MSF ORP, though with the best interest of patients in mind, presented confusion for patients about where to seek care (which partner offered better care?). It also added extra effort from BMOH to coordinate partner activities, run a CTC, and provide security, civil defence and military at Muna Garage. Further, in camps where three different partners operated the three facilities, issues of competition arose:

The only challenge we can clearly point out was the unnecessary competition amongst partners over who gets the glory? Who owns the data? (NGO official).

This competition manifested in many ways including case referrals where MSF France ran ORP in Muna Garage but referred cases to a CTC ran by MSF Belgium in Dala IDP camp. Yet, there were two CTCs in Muna Garage; one ran by BMOH and the other by UNICEF Health. Therefore, it is counter intuitive that cholera patients from Muna Garage were referred to Dala. Equally challenging were patient reluctance to be transferred to other facilities, particularly, when they perceived disagreements between partners. However, one non-governmental organisation reported no issues in referrals or competition as they had an integrated case management setup.

In Dikwa… our health outreach workers referred acute watery diarrhea patients directly to ORPs managed by Family Health International (FHI)360 and from there to CTUs or CTCs equally managed by FHI360. Our strength was not only this in-house referral system but also our willingness to work with other partners (NGO official).

Throughout this study, we documented that challenges with referrals were more common in inter-partner referrals and minimal in intrapartner situations. Other issues with case management were denial of cholera by the locals and lack of qualified pool of people to train for case management.

### Oral cholera vaccination

The vaccination campaign team planned, applied for and deployed OCVs in Borno within 2 weeks as summed up in this excerpt:

With regard to the vaccination… Firstly, we (government, WHO, Global Alliance for Vaccines and Immunization, UNICEF) managed to bring the cholera vaccines in two weeks. Within three days the application was submitted. The next week, it was approved and the next five to six days it came to the country and were shipped to the affected region. Secondly, the polio mechanism, with its good practice and experienced staff, was used for the OCV, and so it was easy to have OCV without … stresses including logistic support. Lastly, well-coordinated structure under the leadership of the EOC, and experienced workforce in conducting immunization…combined together and made it very easy for us to achieve a remarkable accomplishment over the OCV (WHO official).

Additional keys to the OCV deployment within 2 weeks were training at various levels (see online supplementary figure S2), the Lot Quality Assurance Survey that helped know its quality, and high advocacy with the commissioner vaccinating to show OCV is harmless. A broader context further explains the successful deployment of OCV in Borno:

10.1136/bmjgh-2019-002000.supp2 Supplementary data

Between the 31^st^ May and June 1^st^, 2017, Nigeria Center for Disease Control held a cholera preparedness workshop in Abuja where states with history of cholera and international partners were invited. At this workshop, the idea of using OCV in Nigeria was first discussed. … It is important to note that the cholera preparedness workshop was not in response to the outbreak in Borno (Government official).

Two major outcomes of the May/June 2017 workshop were the approval of OCV in Nigeria and start of application process to use the vaccine to fight endemic cholera. Then, the August 2017 Borno outbreak not only gave opportunity to use the newly approved intervention, but also quickly turned an endemic application into an emergency reactive one. A tedious aspect of the vaccination campaign planning is the development of the microplanning guides, but the microplans that had been used in Sierra Leone were adapted for Borno and greatly facilitated this process.

Adaption of the polio platform for OCV had some challenges. First, parents were reluctant to receive OCV as they felt vaccination was for children and not for adults. Polio vaccinators were not familiar with opening OCV vials and so were provided scissors to overcome this difficulty. Vaccination cards were not issued during the first round with the assumption that there will be no second round. Although the latter subsequently became apparent, efforts during the second round to document reception of vaccine during first round proved futile. Poor communication networks hindered data flow from hard-to-reach areas to the central coordination and created anxiety as to whether relevant data would be available to inform needed actions. So supervisors travelled to the villages to get the relevant data. In some instances, partners claimed ownership of data. However, these faded when partners understood that all data belongs to the ministry. Limitations in the polio cold chain and timeliness of vaccine delivery from Abuja to Borno were equally challenging.

We received and immediately moved the vaccines to Borno State through trans-docking because of the storage constraints in Abuja warehouse and because of the policy in the country regarding supplementary vaccines (Government official).

The word transdocking refers to moving inventory from an inbound vehicle directly to an outbound vehicle for transportation without storing in a warehouse. Due to storage constraint, vaccines arrived in batches in Borno and were transdocked to the designated LGAs. Therefore, work-around strategies such as bringing the vaccine in batches in chartered planes and trans-docking were adapted.

As regard vaccine financing, a major challenge was lack of bank accounts at the ward level.

WHO funding system emphasis direct disbursement into beneficiaries’ bank account and works well with those with a bank account, but problematic otherwise. Polio card system works with those without bank account but was not used (WHO official).

Had the polio card system (see online supplementary figure S3), which entails contracting banks to pay on presentation of cards, been adapted, it would have saved the finance team the stress of carrying large sums of money to remote areas for payments. Also the finance team underbudgeted during first round, and workers worked extra days for less pay. Second round budgeting corrected this mistake.

10.1136/bmjgh-2019-002000.supp3 Supplementary data

### Water, sanitation and hygiene

The initial response from UNICEF-WASH was to document living conditions in Muna Garage.

Our immediate outbreak investigation showed several transmission opportunities such as latrines fill-up, overflow (top most risk factors of the outbreak), and collapse; less than half a meter deep shallow latrines inside houses and tents; open defecation in and around refuse dumps; families’ dislike of communal latrines and distance to them; large stagnant pool of water in which people bath, and kids played; and overcrowding in the camp (NGO official).

The above excerpt not only corroborated findings of WHO-led surveillance team, but expanded these to include several faecal oral transmission routes. Of course, if latrines collapsed or overflowed, families will dislike them and turn to open defecation, a practice they are used to in the villages from where they fled. Changing practices of open defecation was a challenge. Likewise complaints about distance to latrines and preference of having less than half a meter-deep latrines in houses/tents were typical of practical ways of circumventing inaccessible communal latrines. Overcrowding in the camp likely forced families to line up to relieve themselves using the limited number of latrines, and this led to their collapse and eventually being non-functional. All these conditions were compounded by stagnant pools of water draining into the camp in which children played. Further, at peak season, electricity from solar panels failed to pump water into communal tanks, so people turned to street vendors. Having identified the transmission routes, UNICEF-WASH (1) provided aqua tabs to households, (2) equipped about 650 volunteers to test water contamination, (3) engaged street water vendors about water safety, (4) promoted good hygiene through social mobilisation, (5) dislodged latrines and (6) chlorinated latrines, water buckets in households and tanks at community level. An unforeseen constraint was reluctance to use chlorine and this led to increased open defecation.

As WASH technicians, we know what to do to curtail an outbreak, but we don’t know exactly what method to adapt. For instance, in our immediate response, we chlorinated water and latrines, but these led to the avoidance of the chlorinated water and latrines resulting in increases in open defecation (NGO official).

Without fully understanding the people’s reluctance to use chlorinated water and latrines, UNICEF-WASH engaged UNICEF Communication for Development (C4D).

…WASH team engaged C4D and in less than two days, they gave feedback as to why there was reluctance to use chlorine, which we failed to recognize. There were stratifications within the camp and community based on faith, language groups, and leadership, which WASH couldn’t initially identify. There was misconception about chlorination. It was rumoured that chlorine was for sterilization, which would stop women from giving birth if they use the chlorinated water. There was also misconception that chlorinated latrines produce chlorine vapor and when women use them the vapor will sterilise them (NGO official).

We note here that the technical people did not use the phrase ‘sterilise water’, which the community could have mistakenly thought to mean sterilise against fertility. What was actually rumoured in the community was:

You see that whitish think that they are putting in water, houses and latrines, it will stop women from giving birth to children (NGO official).

UNICEF-C4D’s mapping to understand the audience by strata, tribe, language, religion and leadership uncovered the misconceptions and rumours about chlorine. This led to deeper understanding regarding, who among these strata do the people trust to talk about promoting chlorine and personal hygiene? Working with UNICEF-C4D, key people in the community were identified who were more effective in transmitting messages that led to chlorine acceptance; It appeared that accepting chlorine required involving and mobilising key people who were trusted. The key people mobilised in the community were ‘Bullemas’, community gate keepers in whom people trust. Attempts to provide interventions in community without consent of ‘Bullemas’ encountered resistance no matter how well intended the interventions were. The UNICEF-C4D staff recognised this, and this intervention was then accepted.

It was noted that the FMWR did not contribute significantly to the outbreak response.

The FMWR intervenes only at the request or when it gets report from the State Ministry of Water Resources of a need for intervention, which was not the case. There is need for UNICEF-WASH to support the Federal Ministry Water Resources, just as WHO does for Nigeria Center for Disease Control, to carry out interventions and the need for the Ministries of Health and Water Resources to work together to combat water borne diseases (Government official).

The State Ministry of Water Resources mentioned above refers to Rural Water Safety and Sanitation Agency (RUWASSA) that was supposed to alert the federal water ministry but did not. In fact, RUWASSA did not act quickly in response to the outbreak in taking leadership role on WASH according to partners. If RUWASSA had notified the Federal Ministry, more support might have been obtained, just as BMOH had from Nigeria Center for Disease Control, so this was viewed as a missed opportunity. The call for UNICEF-WASH to support the FMWR signalled a lack of collaboration between the WASH partners and clearly, the WASH conditions in Muna Garage underscored this need for collaboration. A gap analysis study is needed to inform ways in which these partners should collaborate.

### Cross-cutting themes

#### Risk communications

WHO communication team’s response was a three-pronged approach encompassing (1) risk communication, (2) advocacy/visibility and (3) raising awareness.

We created Outside Broadcasting System, which used speakers to communicate cholera risks as most in affected community did not have access to mass media and electricity. Advocacy and visibility engaged mass media channels while awareness raising distributed flyers addressing Frequently Asked Questions and basic facts about cholera (WHO official).

WHO’s mass media included four international radio stations, one international television (TV), 10 newspaper outlets and internet. The team created pictorial awareness messages using different local languages and engaged a local drama group to influence positive attitudinal and behavioural change.

However, WHO communications used a top down approach and the initial messages were in Hausa. The mobilisers assumed Hausa was spoken widely. Yet, a bottom up approach found Kanuri was spoken predominantly:

The results of our one-on-one communication in the IDP camps indicated that less than 10 people in the camps had radio, that about 12 tribes lived in the camps, and that Hausa was not predominantly spoken as was initially assumed. Thus, our social mobilization was then based in the latter findings, which yielded an understanding of not only the channels and languages of communication but also the content of the messages (NGO official).

Using the bottom up approach, UNICEF-C4D stratified the community by tribe, religion, leadership and languages leading to a change in the main language of communication from Hausa to Kanuri. This approach also uncover deep mistrust of information from government owned and run media. Thus, the first communication’s response was to map community structure to inform response strategies, especially using the more appropriate language.

#### Logistics

The 12 days delay in outbreak declaration delayed the decision to set up CTCs in Muna garage, but when the decision was taken, WHO-Operations Support and Logistics (OSL) had limited time for the task.

In setting up CTCs in Muna Garage, these were needed within less than 4 days, which were timely setup (WHO official).

Although erecting a CTC within 4 days was challenging, more daunting for WHO-OLS was mapping inventory between government and partners to avoid stock shortages. While some partners were hesitant to share inventory availability, others lacked the data. Mapping the supplies, however, did uncover critical shortages in Ringer’s lactate, which could not be purchased locally, and this prompted WHO-OSL to act assertively to import the product. It was not clear why Ringer’s lactate could not be sourced locally. The most essential action for logistics was joint planning meeting and collaboration between National Strategic Cold Store, BMOH, WHO, manufacturers and the transport agents to ensure that the right supplies were at the right place in the right quantities and at the right time.

#### Coordination

The health sector-coordinating unit at BMOH coordinated all emergency response activities with two main objectives; avoid duplication of effort and ensure data harmonisation.

Coordination meant coordinating partners such as WHO, MSF, FHI360, UNICEF, and Alliance for International Medical Action to ensure no duplication of effort. It also meant ensuring that in Areas where two or more partners worked, data coming out were harmonized; not data from UNICEF or WHO or MSF (Government official).

To illustrate, the same patient might visit treatment centres operated by different partners with the potential for double counting. So partners had to report only their respective contributions to patients they treated. A major challenge was coordinating budget between government and partners at the start of the outbreak response.

It was difficult to get what was needed (budget) from and available with partners and what the government needed to contribute (Government official).

Hurdles in budget allocation spilled over into partner hesitancy during team formation; some were reluctant to be part of a team and others did not register with the government. Competing demands between insecurity and health emergencies, lack of synergy between WASH and health sectors compounded the situation:

WASH was doing its own activities and so was WHO (Government official).

As the outbreak continued, the need to overcome coordination difficulties became critical; thus, the handing over of the EOC by WHO to BMOH just at the onset of the outbreak response was very important.

The greatest success in overcoming coordination hurdles was the EOC, which was used as the planning and response center for all partners. The second was the State identification of the key thematic pillars and populating these with key partners (Government official).

EOC made it possible to coordinate all response activities including implementation of the Incident Management Structure and inter-sectorial multiagency meetings held at 16:00 hours daily.

Until declaring the State free from cholera, there was joint government and partner meetings at the EOC at 4pm daily where every team lead debriefed others of the situation on the field and progress made. (WHO official).

During the 16:00 hours daily meetings, epidemiological curves and geographical coordinate maps were presented to direct multisectorial teams to priority locations. Overall, both partners and government concurred that a well-functioning EOC was essential to overcoming coordination hurdles.

## Discussion

This qualitative study explored perspectives about the emergency response to the 2017 cholera outbreak in Borno, Nigeria. This was the first time that Nigeria included OCV (in reactive context) as part of their response to a cholera outbreak.^24^ The campaign was initiated within 2 weeks from the time of application and targeted 891 137 people at least 1 year in six LGA.^25^ Similar to Iraq,^6^ a key to this laudable success was the robust polio infrastructure in Borno, whose budget blue prints, target populations and vaccinators were adapted. In addition, OCV microplanning guides developed in Sierra Leon were imported and adapted to suit the Borno context, and this exemplifies the importance of international networking in cholera prevention and control. The preparatory workshop hosted by the Nigeria Center for Disease Control in Abuja in May/June 2017 led to the OCV approval and subsequent registration for use in Nigeria that same year; this was critically important to the campaign later in the year. Also important was the advocacy that led to the Commissioner of Health publicly taking the vaccine to show it is safe.

Mass communications were needed to dispel anxiety, rumours about and parental reluctance to OCV in Borno, consistent with findings in other settings.^26^ Yet, these communications did not immediately change reluctance to accept OCV; however, when cholera deaths occurred, people changed their minds and took the vaccine. Had formative research been conducted prior to OCV campaign, this may have provided information on the behavioural determinants of OCV acceptance; thus, there is urgent need for formative research as part of Monitoring and Evaluation of OCV and this should be initiated prior to the campaign to address issues of vaccine reluctance prior to OCV distribution.

Finally, yet importantly, although the polio microplans were used, its card mode of payment was not included in the case of OCV. If it had been used, this would have simplified paying vaccinators without bank accounts. This element should be used in future OCV campaigns.

We found that epidemiological surveillance (EWARS,^19^ IDSR^23^ and the phone platform) quickly detected and notified health authorities of the outbreak. The latter also classified the index case as fitting WHO’s standard case definition,^24^ but confirming the outbreak with a positive culture delayed the response. According to WHO policy,^24^ a positive RDT result must be confirmed by culture to declare a cholera outbreak and this normally takes about 2 days.^27^ In this case, a positive RDT result was not confirmed by culture in the local UMTH laboratory, but was subsequently confirmed in the reference laboratory in Lagos 10 days later. The time lag between the period of waiting for culture confirmation created room for the wide spread of the disease.

Thus, the false negative culture at UMTH laboratory delayed outbreak confirmation and declaration. The delayed declaration highlights the urgent need for increasing lab capacity at the state level in Nigeria and/or use the RDTs in a manner that will allow for a declaration sooner. The need for such improvements is further illustrated by our finding that fear of political repercussions hindered this declaration even with strong clinical evidence of transmission and positive RDT result.

Our data suggest that sewage from a leaking latrine likely amplified the 2017 Borno outbreak. Although authorities were alerted in a timely manner, the leaking latrine was still unrepaired 1 week later. The delayed establishment of the CTC in Muna Garage^28^ because of disagreements between government and partners, were missed opportunities to prevent cholera spreading out of the camp. As found elsewhere,^29^ the Muslim festival, Eid El Kabir, which involves much food sharing and population movement that coincided with the outbreak onset, was not appreciated. This suggests that national cholera plans should include specific guidelines with regard to handling religious festivals and funerals during outbreak emergencies.

The outbreak response from the RUWASSA was slow despite the presence of high-risk channels of cholera infection such as overflowing/non-functional/collapsed latrines, open defecation,^30^ overcrowding in IDPs camp^31^ and stagnant water in which children played; these high-risk channels have been linked with rapid spread of cholera.^32–35^ Conducting a gap analyses would inform RUWASSA in ways to improve WASH conditions in camps. The call from the FMWR requesting UNICEF to assist water ministries to carry out their duties, underscores the need for this gap analysis.

There was need to strengthen intracollaboration and intercollaboration between water and health ministries to improve future response to emergencies. Importantly, within UNICEF, two groups were not communicating initially, UNICEF-WASH and UNICEF-C4D, but their later coordination led to the importance of consultation with ‘Bullemas’ (village headmen). This engagement of the community and with communication experts was critical.^36^ Community entry^37 38^ without community involvement^39 40^ have been shown in different contexts to lead to community resistance,^41 42^ and even, to physical violence.^43 44^

To the credit of the case management teams, the CFR was 1.14%; this is lower than seen in previous outbreaks in Nigeria,^17^ but is still a bit higher than the 1% as recommended by WHO.^24^ Several issues could have impacted the increased numbers of deaths including (1) delayed declaration of outbreak, (2) initial attempts to manage the outbreak within the normal health facility, (3) disagreements between government and partners), (4) competition between partners, (5) patient reluctance to referrals, (6) denial of cholera by the locals and (7) insecurity posed by Boko Haram (hindered 24 hours operation of CTUs/CTCs.^45–47^). Of the 61 deaths reported, 56 died at a health facility (it is possible that others may have died but were not reported.). Appropriate studies are needed to establish the links between each of these observations and cholera deaths.

A very critical component of the response was risk communications. In the three-prong approach WHO used for messaging,^48^ a theatre group was intended to reach the local population. However, this lacked community input to inform the choice of language, and so was rather seen as top down communication. When UNICEF-C4D used a bottom up approach^36^ (first mapping the community structure), the language changed from Hausa to Kanuri, and the main channel of communications shifted to ‘one-on-one.’ This illustrates that a bottom up approach is critical for evidence-based decision making in resource scarce settings.

The logistics team’s response was very appropriate. From setting up CTCs^47^ to mapping inventory and to maintaining the OCV could chain, the logistics team ensured the right products or services in the right numbers at the right place for the right price and at the right time.^49^ Logistic challenges included partner reluctance to release stock availability for planning, and inability to source Ringer’s lactate locally. The loopholes in logistics plans which became apparent during this outbreak indicates that logistics were not given due attention in anticipation of an outbreak; and thus, practice drills are needed to inform the practicality of logistics plans.

The lack of gaps, overlaps and duplications in the outbreak response suggests that coordination was largely effective. For instance, double counting, if allowed would have led to bias in the CFR or attack rates. As has been underscored in a systematic review,^50^ the well-functioning EOC was key to effective coordination.^51^ It enabled BMOH to take leadership, ownership and overcoming hurdles in budget allocation, hesitancy in team formation, competing demands between insecurity and health, and correcting the lack of synergy between WASH and health sectors. The EOC was the hub for coordinating strategic decision making based on the epi curves and geographical coordinate maps that were shared between all emergency response teams at 16:00 hours daily. Yet, there was controversy between the EOC^51 52^ and United Nations cluster^53^ approaches to coordinating the emergency response. However, this topic is beyond the scope of this study, but one that needs evaluation to understand the conflict.

In sum, quantitative studies have analysed humanitarian crises-related cholera intervention measures in isolation focusing on sanitation^54 55^ in refugee camps, water/hygiene/treatment,^56 57^ and OCV^6–10 58^ in IDP camps. Without down playing the rigour of these quantitative studies, excepting Spiegel *et al,* 11 qualitative studies are needed to explore comprehensive cholera emergency response measures in humanitarian crises including IDPs camps, WASH, case management, surveillance, OCV, coordination, logistics and communication.

## Conclusion

### Surveillance

Strengthening of laboratory capacity to rapidly confirm cholera cases is urgently needed to confirm outbreaks. While building laboratory capacity is needed over the long term, a cholera outbreak may need to be declared and a full-scale response should be initiated based on evidence of clinical transmission supported by multiple positive RDTs. This declaration should involve local leaders such as village headmen as well as Ministry officials.

### Water, sanitation and hygiene

Clearly, improved WASH conditions in the IDP camps including functioning boreholes, wells and latrines were needed to prevent outbreaks. To avoid misconceptions, misunderstandings and rumours, technical WASH experts need to coordinate with communication experts prior to community interventions.

### Case management

Partners providing case management should ensure that patient care is the primary goal and avoid self-promotion (who owns data? or who gets the glory?). For outbreaks like this one, the establishment of ORPs, CTUs and CTCs are highly recommended rather than attempting to care for the many patients using existing primary healthcare facilities.

### OCV

OCV was obtained rapidly after the application was submitted. The polio vaccination infrastructure was highly effective in implementing OCV for the first time in Nigeria. However, the polio card mode of payment of staff without bank accounts should be used for OCV as well. To overcome vaccine reluctance, formative research should be conducted prior to OCV roll out as part of Monitoring and Evaluation.

### Risk communications

An early response to an outbreak should be mapping the community structure to inform interventions including choice of language and communication channels (TV, tadio, one on one). In resource-poor settings, a bottom up approach should be used to build networks, trust and contacts with local leaders.

### Logistics

Prior to the outbreak, no regular meetings were held between partners and logistic managers to assess inventory to avoid stock outs, but were held during the outbreak. For the future, this should continue and it will be wise to improve logistics preparedness by conducting practice drills or simulations of logistics plans to ensure practical feasibility.

### Coordination

The EOC was key in strengthening coordination of the emergency response of the outbreak. Epidemiological curves and geographic coordinate map presentations during meetings at EOC at 16:00 hours daily proved highly helpful in coordinating the response. On the other hand, coordination of WASH response was slow. A more active role for RUWASSA is needed to coordinate the WASH sector response. This includes taking ownership and leadership of WASH response in the State and close synergy with the FMWR and with UNICEF who also should play a critical role.

Conclusively, all partners should understand that the government is in charge, but needs their support to respond to emergencies. In addition, government should ensure that all camps are officially recognised, establish communication channels between partners with a unified approach to the emergency response. Finally, partners need to put beneficiaries’ interest first over partner interest, which is critical to dealing with emergencies.
