# Supplementary material for: The multi-sectorial emergency response to a cholera outbreak in Internally Displaced Persons camps in Borno State, Nigeria, 2017
Source: BMJ Glob Health. 2020 Jan 28;5(1):e002000. doi: 10.1136/bmjgh-2019-002000 (PMC7042583; doi:10.1136/bmjgh-2019-002000)
Supplement: Supplementary data [file bmjgh-2019-002000supp001.pdf]

S3 Supplement: Outbreak response questionnaire

Qualitative Interview to Understand Cholera Emergency Response in Borno State, Nigeria

Good morning / good afternoon. My name is _____________ and I am with WHO Geneva. We are attempting to learn more about the response to the 2017 cholera outbreak in Borno Sate, Nigeria.

The objective of this oral interview is to understand your perspectives as someone who represented your organization in the planning and implementation of outbreak response, which incuded oral cholera vaccination (OCV) as part of the cholera outbreak response. The interview will take about thirty minutes.

Your participation is voluntary and you can choose to stop or skip any questions at any time. The information I collect during this interview will be audio recorded and be used in a report for the Ministry of Health. It can be attributed to you by name and organization, by the type of organization you represent (government, NGO, UN), or it can be anonymous. If you are attributed by name and organization, please note that you can go "off the record" at any point so we treat that information as an anonymous source. How would you like to be identified for this interview (name and organization, type of organization, or anonymous)? Please let me know if you would like to change this at any time during interview.
If you are willing to consent to this interview, please verbally state your consent by stating "I consent to this interview".

1. **<u>Interviewee information and general questions</u>**
    a. Name, organization, type of organization (goverment , non-gernmental organization, United Nations)
    b. Professional experience and background
    c. What was your role of ------- in the cholera outbreak response?
    d. How did you learn of the outbreak? What did you do after learning about the outbreak?
    e. Was the declaration of the outbreak by the Borno Ministry of Health timely?
    f. What outbreak response related activities did your organization support?
    g. Where did you support outbreak response related activities?
    h. Can you briefly describe the outbreak response successes and challenges?
    i. How were special events like religious or national festivals handled during the outbreak response?

2. **Knowledge regarding OCV (OCV-specific questions)**
    a. Where did you support OCV activities (or planning)?
    b. Have you implemented or supported OCV in other cholera outbreaks in the past?
    c. Can you describe the level of knowledge and/or gaps in knowledge about the use of OCV during the outbreak response?
    d. In Nigeria, who drove the decision to consider OCV?
    e. What were the major considerations for using OCV in the response?
    f. Were there concerns about safety of the vaccine?
    g. What opportunities did you have in responding to the outbreak? How were those opportunities used?
    h. How was financing of the OCV response handled? Specifically, how were local level staff paid?
    i. Were there special events planned during the OCV campaign? How were these events managed?

Ngwa MC, *et al. BMJ Global Health* 2020; 5:e002000. doi: 10.1136/bmjgh-2019-002000

3. **Coordination among actors in outbreak response: health, WASH, and nutrition**
    a. With the inclusion of OCV, how were partners coordinated to an integrated response? i.e., coordinated health (OCV, case management, surveillance, lab, M&E, logistics), WASH (chlorination, logistics, etc.), communication (community mobilization/sensitization, risk communication, logistics, etc.), and nutrition (community mobilization, supplementary feeding program, Outpatient Therapeutic Program, Stabilization Center/Inpatient care) sectors.
    b. How were WASH partners brought into the discussion of OCV?
    c. Were there workshops to discuss and plan for the use of OCV? When and where did they happen? Were these efforts effective at bringing partners together?
    d. As OCV has not been used in Nigeria before, how was it included in the national cholera control plan to be used here?
    e. How did the security situation affect the ability to deliver an effective response?
    f. Were preparedness plans in place before the outbreak? If yes, was OCV part of that preparedness plan? If no, why?

4. **Risk communication**
    a. Can you describe the WASH situation in the IDP camps prior to the outbreak?
    b. What assessment and/or epidemiological data were considered to support the OCV application?
    c. Did you contribute to the risk assessment?
    d. How was the risk assessment used to guide micro planning?
    e. What risk factors for cholera were taken into account?
    f. How was the risk assessment translated for decision-makers? (i.e., Senior MOH, WHO, etc.)
    g. In your perspective, how was the financing of the OCV handled?
    h. Was the surveillance data strong enough to support reactive use of OCV?

5. **Stockpile application and process**
    a. How long did it take to develop and submit the application to the Stockpile?
    b. Who was involved? Who drove the process? (i.e., WHO, MSF, NCDC, NPHCDA, LGA/ State MOH, etc.)
    c. Did the NCDC or NPHDA or LGA or BMOH have the qualified personnel (i.e., persons with OCV knowledge) to make crucial decisions about the stockpile?
    d. What were the major challenges/barriers to completing the application?
    e. Was the anticipated coverage of the campaign appropriate for the phases of the outbreak?
    f. Were there any mistakes made in the consideration of the OCV response?

6. **Wrap-up**
    a. Was technical leadership from WHO and the government (NCDC, NPHDA, BMOH, LGAs etc.) for this intervention appropriate for the complex operating environment with Boko Haram?
    b. Did the decision-makers (especially at the BMOH level) have adequate, accurate information, knowledge and data to support an OCV campaign?
    c. What are the lessons learnt?
    d. Any general observations that we have not considered?
    e. Can you provide any monitoring and evaluation (M&E), financial, cost or recorded data and any further information in support of your organization’s response to cholera?

Ngwa MC, *et al. BMJ Global Health* 2020; 5:e002000. doi: 10.1136/bmjgh-2019-002000
