# Supplementary figures and images for: The multi-sectorial emergency response to a cholera outbreak in Internally Displaced Persons camps in Borno State, Nigeria, 2017
Source: BMJ Glob Health. 2020 Jan 28;5(1):e002000. doi: 10.1136/bmjgh-2019-002000 (PMC7042583; doi:10.1136/bmjgh-2019-002000)

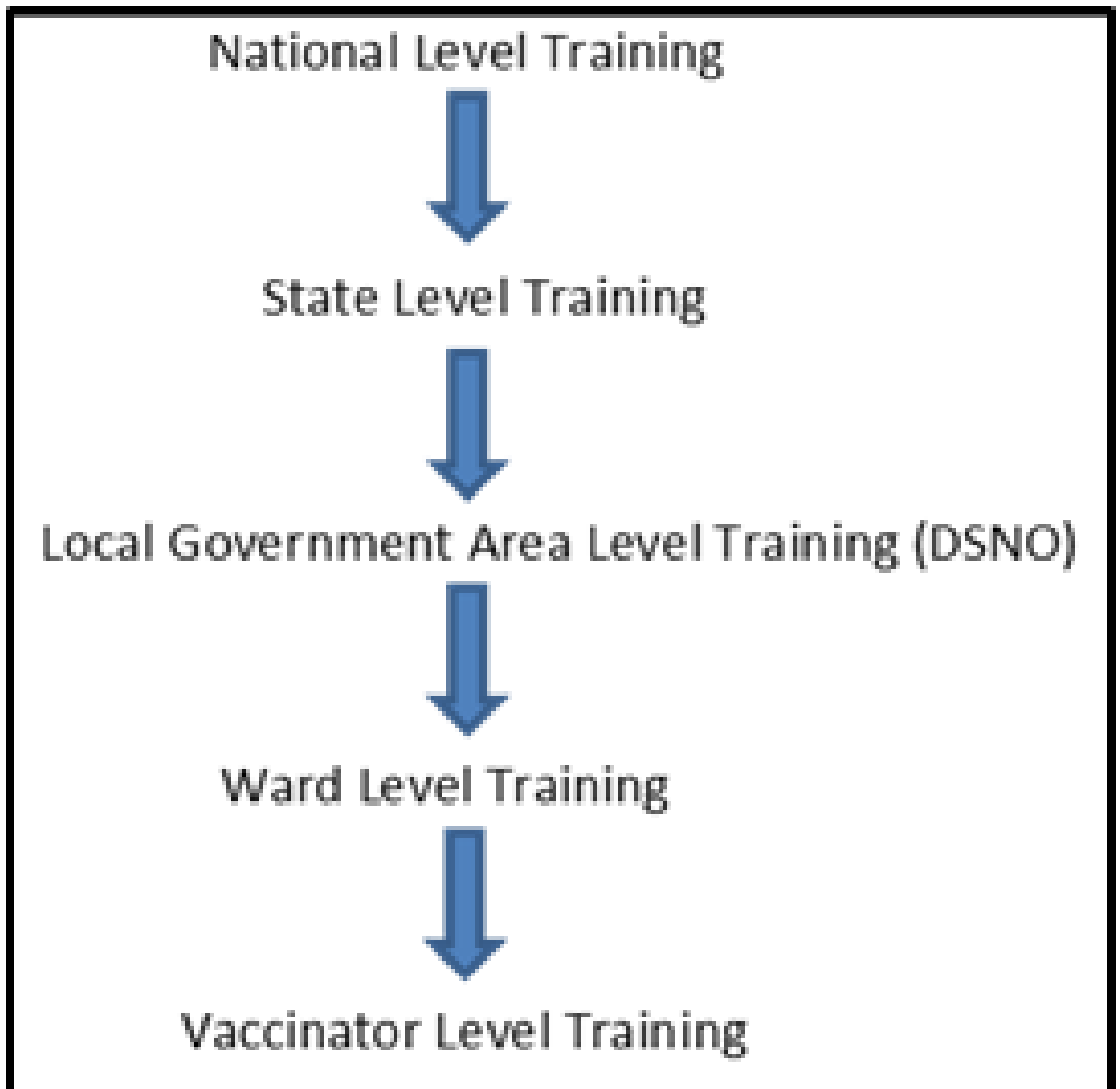

Supplement: Supplementary data [file bmjgh-2019-002000supp002.pdf]
