# Supplementary material for: The multi-sectorial emergency response to a cholera outbreak in Internally Displaced Persons camps in Borno State, Nigeria, 2017
Source: BMJ Glob Health. 2020 Jan 28;5(1):e002000. doi: 10.1136/bmjgh-2019-002000 (PMC7042583; doi:10.1136/bmjgh-2019-002000)
Supplement: Supplementary data [file bmjgh-2019-002000supp003.pdf]

Polio

218

Ward Focal Supervisor Activity Card (WFSAC) **Level 3** **OCTOBER 2017 SIPDs - NIGERIA**

Immunization Plus Days (IPDs) Implementation Date:..			PLEASE AFFIX PHOTO HERE
STATE:	LGA:	WARD:	
Name of Ward Focal Supervisor:	Signature of Ward Focal Supervisor:	DISTRICT:	
Your Own Mobile Phone Number:	Date of Birth:	Name of LGA Team Member:	
Personal Bank Account Number:	Name of Bank:	Signature of LGA Team Member:	

Description of activity	Signature of LGA Team Member:	Description of activity	Signature of LGA Team Member:
IPDs day 1 @ N 1,300.00		IPDs day 5 @ N 1,300.00	
IPDs day 2 @ N 1,300.00		IPDs day 6 @ N 1,300.00	
IPDs day 3 @ N 1,300.00			
IPDs day 4 @ N 1,300.00			
		Transport allowance	

This section for Bank use only:

Date of Disbursement:________________ Amount Disbursed:________________

Name of Paying Officer — Signature of Paying Officer

Name of LGA Team Member — Signature of LGA Team Member

Name of Payee — Signature of Payee
